# Winning and Losing: Effects on Impulsive Action

**DOI:** 10.1037/xhp0000284

**Published:** 2016-11-03

**Authors:** Frederick Verbruggen, Christopher D. Chambers, Natalia S. Lawrence, Ian P. L. McLaren

**Affiliations:** 1 Department of Psychology, University of Exeter; 2 Department of Psychology, Cardiff University; 3 Department of Psychology, University of Exeter

**Keywords:** cognitive control, gambling, impulsive action, sequential effects, emotion

## Abstract

In the present study, we examined the effect of wins and losses on impulsive action in gambling (Experiments 1–3) and nongambling tasks (Experiments 4–5). In each experiment, subjects performed a simple task in which they had to win points. On each trial, they had to choose between a gamble and a nongamble. The gamble was always associated with a higher amount but a lower probability of winning than the nongamble. After subjects indicated their choice (i.e., gamble or not), feedback was presented. They had to press a key to start the next trial. Experiments 1–3 showed that, compared to the nongambling baseline, subjects were faster to initiate the next trial after a gambled loss, indicating that losses can induce impulsive actions. In Experiments 4 and 5, subjects alternated between the gambling task and a neutral decision-making task in which they could not win or lose points. Subjects were faster in the neutral decision-making task if they had just lost in the gambling task, suggesting that losses have a general effect on action. Our results challenge the dominant idea that humans become more cautious after suboptimal outcomes. Instead, they indicate that losses in the context of potential rewards are emotional events that increase impulsivity.

Scientists often attribute adaptive and goal-directed human behavior to a cognitive control system that organizes, monitors, and alters the settings of lower-level cognitive processes. This allows people to suppress or replace impulsive, habitual, or inappropriate actions. For example, cognitive control is required to quickly suppress prepotent actions when a stop or no-go signal is presented (Miyake et al., 2000; Ridderinkhof, van den Wildenberg, Segalowitz, & Carter, 2004; Verbruggen & Logan, 2008). Cognitive control is also required for responding with restraint (without completely suppressing all ongoing responses). For example, people slow down and respond more cautiously when they expect a stop signal to occur (Verbruggen & Logan, 2009b; for reviews, see, e.g., Aron, 2011; Stuphorn & Emeric, 2012). This slowing can be attributed to top-down control adjustments in attentional settings to enhance detection of the stop signal, and adjustments in response or motor settings to prevent premature responses (e.g., Elchlepp, Lavric, Chambers, & Verbruggen, 2016). Similarly, the cognitive control system can attenuate motor activation in situations in which accurate responses are required (e.g., Forstmann et al., 2008) or when there is uncertainty about which action has to be selected (e.g., Frank, 2006).

Many psychological theories assume that the cognitive control system also alters the settings of lower-level systems when people make an error or when outcomes are otherwise less desirable than anticipated (Egner, 2008; Ullsperger, Danielmeier, & Jocham, 2014; Verbruggen, McLaren, & Chambers, 2014). For example, people often slow down after they make an error (“posterror slowing”; Laming, 1968; Rabbitt & Phillips, 1967; Rabbitt & Rodgers, 1977). Such sequential effects have been observed in a variety of tasks, and are usually attributed to the cognitive control system: It monitors for errors (Alexander & Brown, 2010; Brown & Braver, 2005; Taylor, Stern, & Gehring, 2007), the occurrence of conflict between choice options (Botvinick, 2007; Botvinick, Braver, Barch, Carter, & Cohen, 2001; Yeung, Botvinick, & Cohen, 2004), or more generally, events that are worse than expected (Botvinick, 2007; Hajcak, Moser, Holroyd, & Simons, 2006; Nieuwenhuis, Yeung, Holroyd, Schurger, & Cohen, 2004). When the control system detects such events, it adjusts the parameters of task-relevant processing pathways. These adjustments usually increase response latencies (i.e., people become more cautious) but can reduce the likelihood of further negative or suboptimal outcomes on subsequent trials (e.g., Dutilh, Vandekerckhove, et al., 2012; Purcell & Kiani, 2016).

The present study examined sequential effects of wins and losses in a gambling task. Gambling is a recreational activity that many people engage in (e.g., 73% of the U.K. adult population; Wardle et al., 2011), but for a minority of people, it can turn into pathological or problematic behavior, which may have serious adverse personal and social consequences (Clark & Limbrick-Oldfield, 2013; Potenza, 2014). Problem gambling has been associated with impairments in cognitive control. For example, the control system may be required to regulate risk taking by suppressing superficially attractive but risky options (e.g., Billieux, Gay, Rochat, & Van der Linden, 2010; Cohen & Lieberman, 2010; Knoch et al., 2006; Trepel, Fox, & Poldrack, 2005; Verbruggen, Adams, & Chambers, 2012). Cognitive control may also be required to adjust decision-making strategies after a loss or a suboptimal outcome, and failures to do so may contribute to the development of problematic behaviors, including problem gambling (Brown & Braver, 2008; Garavan & Stout, 2005; van Holst, van den Brink, Veltman, & Goudriaan, 2010).

Cognitive control accounts predict decreased gambling after a loss than after a win. However, people often *gamble more after a loss* than after a win (Clark, 2010). Similar effects of negative outcomes on choice behavior have been observed in a variety of tasks and situations, including the stock market (e.g., Imas, 2014; Smith, Levere, & Kurtzman, 2009). Many explanations have been proposed for such sequential effects (for a short review, see Smith et al., 2009). For example, according to prospect theory (Kahneman & Tversky, 1979), the subjective value of an option can be influenced by immediately preceding gains and losses (Smith et al., 2009). This could explain why people gamble more after a loss than after a win, and attempt to recover previous losses.

Cognitive control accounts also predict slower responding (increased response caution) after a loss than after a win. By contrast, several gambling studies found that subjects were *faster* to initiate the next gamble *after a loss* than after a win (Corr & Thompson, 2014; Delfabbro & Winefield, 1999; Dixon, MacLaren, Jarick, Fugelsang, & Harrigan, 2013; Shao, Read, Behrens, & Rogers, 2013; but see also Brevers et al., 2015; Goudriaan, Oosterlaan, de Beurs, & van den Brink, 2005). This finding indicates that negative outcomes in a gambling task can lead to impulsive (fast emotionally charged) actions, and challenges the dominant idea that humans become more cautious after suboptimal outcomes. However, many gambling studies lack a proper baseline, so it is unclear if shorter response latencies after a loss than after a win are due to altered performance after a loss, altered performance after a win, or some combination of the two. In other words, without a baseline, we cannot be sure if the sequential effects of gambling are inconsistent with the cognitive control accounts. Another complicating factor is that the feedback after a win is usually different from feedback after a loss. For example, wins are often associated with extra sounds or additional visual stimuli (e.g., Delfabbro & Winefield, 1999; Dixon et al., 2013). The present study will control for these perceptual factors and explore if and how outcomes in a gambling task influence initiation times on subsequent trials.

## Overview of the Experiments

In the present study, we examined how wins and losses influence impulsive action in both gambling (Experiments 1–3) and alternating nongambling tasks (Experiments 4–5). Many judgment and decision-making studies have examined how gains and losses influence choice (see above). However, response latencies are often ignored in the judgment and decision-making literature, even though they can provide useful information about how people react to positive and negative events. Our main aim was to address this gap in the literature. Therefore, the present study focuses on response latencies. We will provide a general overview of the choice data after the final experiment, but an extensive overview of the very rich and extensive judgment and decision-making literature is beyond the scope of this paper.

**Figure 1 fig1:**
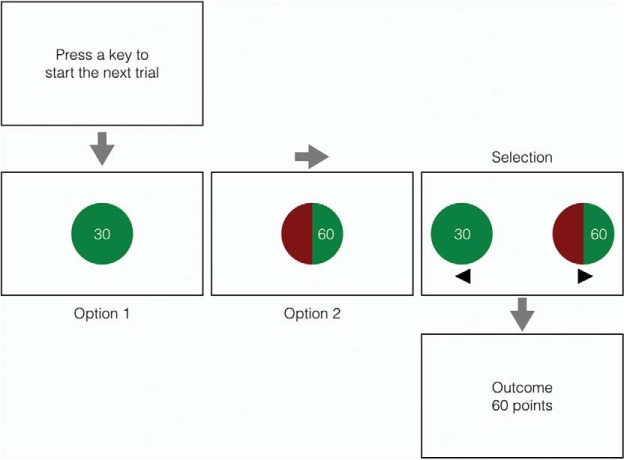
An example of a trial in the gambling task. Each trial commenced with a start message. Then we presented two options successively for 1 s. The first option always represented a guaranteed amount of points that was awarded when subjects did not gamble. The second option always represented a higher amount, but here, the probability of winning was less than 100% (the gamble). The exact probability of winning was indicated by the areas in the “pie chart.” The green area represented the probability that subjects could win the amount shown in the chart, and the red area represented the probability that they would get nothing. Then the options were presented together and subjects indicated whether they wanted to gamble or not by pressing the corresponding arrow key (there was no time limit). After subjects indicated their choice, feedback was presented for 1 s—in this example, the subject elected (successfully) to risk a certain 30 points on a 50:50 gamble to win 60 points (see Method section under Experiment 1 for further details). See the online article for the color version of this figure.

In Experiment 1, we piloted our gambling task (see Figure 1), which is similar to tasks that have been used to measure the neurocognitive correlates of decision-making under risk (e.g., De Martino, Kumaran, Seymour, & Dolan, 2006; Tymula et al., 2012), and the influence of emotion and motivation on economic choices (e.g., Breiter, Aharon, Kahneman, Dale, & Shizgal, 2001; Ernst et al., 2004; Mellers, Schwartz, & Ritov, 1999; Wright, Morris, Guitart-Masip, & Dolan, 2013). Subjects played a very simple game in which they had to win points. Each trial started with the presentation of an amount of points that was guaranteed if subjects decided not to gamble. This was followed by the presentation of a gamble, which was associated with a higher number of points, but the probability of winning the gamble was lower than 100%. After subjects indicated their choice (i.e., to gamble or not), we presented the outcome of the trial: After a gamble, feedback indicated whether subjects had won points (“gambled win”) or not (“gambled loss”); when subjects did not gamble, they kept the number of points shown at the beginning of the trial (i.e., the guaranteed amount associated with the nongamble option). The nongambling trials are our baseline to determine whether gambled losses, gambled wins, or both influenced action. Importantly, apart from the (inevitable) differences in amount, there were no perceptual differences in feedback between the various trial types (i.e., nongamble, gambled loss, and gambled win).[Fig-anchor fig1]

In Experiments 2a and 2b, we also acquired subjective ratings on half of the trials to further assess motivation and subjective beliefs (Clark et al., 2013). In Experiments 3a and 3b, we manipulated the background of the screen to explore the stimulus- or context-specificity of the sequential effects of losses and wins. Finally, Experiments 4 and 5 examined the effect of gambling on response latencies in a subsequent “neutral” decision-making task in which subjects could not win or lose points.

We measured the initiation time of the next trial (start reaction time [RT]) in each experiment. In other words, we examined how quickly subjects acted after a win or a loss. For completeness, we present an overview of the choice data at the end of the manuscript. To summarize the main findings of the choice analysis: In all experiments, subjects gambled on approximately 50% of the trials (but there were large individual differences; see Supplementary Materials). Importantly, they gambled less after a gambled win than after a nongamble, and the probability of gambling was highest after a gambled loss. These findings are consistent with previous studies (see above), and indicate that choice can be influenced by the outcome of the previous trial. The main question we address below is whether gambling outcomes also influenced impulsive action.

## Experiment 1

### Method

#### Subjects

Twenty students (eight males; age: *M* = 19.9 years, *SD* = 1.4) from the University of Exeter participated for monetary compensation (£5) or partial course credit, plus money won in the gambling task (see below). Two subjects were replaced because they rarely gambled in the task; consequently, there were not enough trials (*N* < 5) for the sequential analyses. The subject exclusion criteria and target sample were determined before data collection, based on another pilot study (*N* = 20) in which we found large effects of the outcome of a gamble on the start RT of the next trial (Cohen’s *d*_z_ between .7 and 1.2). All experiments of the present study were approved by the local research ethics committee at the School of Psychology, University of Exeter. Written informed consent was obtained after the nature and possible consequences of the studies were explained.

#### Apparatus and stimuli

The experiments were run on a 21.5-in. iMac using Psychtoolbox (Brainard, 1997). We used a standard QWERTY keyboard for response registration. On each trial, two choice options were represented by pie charts (diameter: 5 cm) containing a certain amount of points (see Figure 1). The first option always represented an amount of points that was guaranteed if subjects decided not to gamble. The second option always represented the gamble. If selected, subjects could risk the guaranteed amount to win a higher amount of points; however, the probability of winning the higher amount was always less than 100%. The exact probability of winning was indicated by the areas of the pie chart. A green (RGB: 0-127-0) area represented the probability that subjects could win the amount shown in the pie chart; a red (RGB: 127-0-0) area represented the probability that they would get nothing. Thus, the larger the green area, the higher the probability of winning. The red segment was always on the left within the “gamble” pie (see Figure 1). The options were presented against a white background (RGB: 255-255-255). The amounts appeared in the center of the green area of the pie chart (font: Arial; font size: 30 point; font color: white).

The amounts and the gambles were randomized across trials: the amount associated with the nongamble (i.e., the guaranteed amount) varied between 20 and 50 (i.e., 20, 30, 40, 50), and the amount associated with the gamble was 1.5, 2, 3, or 4 times higher than the guaranteed amount. This resulted in 16 possible combinations (see Appendix A). The probability of winning the gamble varied between .25 and .66 (i.e., .25, .33, .50, .66), and was adjusted to keep the expected value of the gamble and the nongamble the same (e.g., when the guaranteed amount for the nongamble was 50, and the gamble was 200, then the probability of winning the gamble was .25; 50 = .25 × 200; see Appendix A).

#### Procedure

Figure 1 gives an overview of the trial structure. Each trial commenced with the message “Press a key to start the next trial.” After subjects had pressed any key of the keyboard and 500 ms had elapsed since the presentation of the message,^1^ the guaranteed amount associated with the nongamble was presented in a green pie chart. Then the gamble was presented in a red-and-green pie chart. Both pie charts were presented in the center of the screen for 1 s. After they had been presented separately, subjects saw them again together (one on the left and one on the right of the screen; distance between the two options: 5 cm). At this point, they had to decide whether they wanted to gamble or not by pressing the left- or right-arrow key of the keyboard for the left or right option, respectively (see Figure 1). At the beginning of the experiment, subjects were informed that they were guaranteed to earn points if they did not gamble. There was no time out during the choice phase. Left and right arrows were presented below the options to remind subjects that they could respond only at this stage, and they were told at the beginning of the experiment that they could use the index and middle finger of their right hand to press the keys. The location of the nongamble and gamble options (i.e., left or right of the center of the screen) was randomized across trials.

After subjects selected an option, the computer showed the outcome of their choice. If they had selected the gamble, the computer indicated whether they had won the points indicated in the pie chart (gambled win; e.g., “outcome = 200 points”) or not (gambled loss; “outcome = 0 points”). To determine the outcome of a gamble, the computer selected a random number between 0 and 1 on each trial, and subjects had won the gamble if the selected number was smaller than *p*_win_. If they had selected the nongambling option, subjects always received the guaranteed amount shown at the beginning of the trial (e.g., “outcome = 50 points”). Finally, an error message was presented if subjects pressed an incorrect key (“Incorrect response. Use the left and right arrow keys.”); they did not get any points on such trials. After 1 s, the next trial started with the “Press a key to start the next trial” screen.

To ensure that there were enough trials for the sequential analyses, the gambling task consisted of 256 trials. For most subjects, the experiment lasted 20–30 min. Subjects were told that if they wished to do so, they could take a short “minibreak” between trials, as there were no fixed breaks after a set number of trials. Consistent with previous research (e.g., Tymula et al., 2012), we made choices consequential: At the end of the experiment, the computer randomly selected the outcomes of 10 trials. The sum of these was converted into real money: for every 100 points, subjects got £1 extra. The maximum additional payout was £5 (range: £0–5). Subjects were informed about the payoff structure at the beginning of the experiment.

#### Analysis

All data processing and analyses were completed using R (R Development Core Team, 2015). All raw data files and R scripts used for the analyses are deposited on the Open Research Exeter data repository (http://hdl.handle.net/10871/17260).

In the sequential analyses, we distinguished between trials that followed a nongamble (our baseline), trials that followed a “gambled” win, and trials that followed a “gambled” loss. For each trial type, we calculated how quickly subjects started the next trial (start RT). Due to the payoff manipulation (i.e., at the end of the experiment, the computer randomly selected the outcome of 10 trials), points did not accumulate over the course of the experiment. Therefore, we did not control for overall accumulation of points in the analyses, but we show in Supplementary Materials that the effect of trial outcome on start RT was similar in the first and second half of the experiment. Because some subjects did not gamble often, we could not explore whether a loss or win occurred in a context of doing well or in a context of doing poorly (see, e.g., Mellers, 2000, p. 915).

We excluded trials on which start RT was above 5,000 ms or the latency of the choice response (i.e., the left/right arrow response) was above 2,500 ms, trials on which subjects pressed an incorrect key, and trials that followed such incorrect trials. This resulted in a data exclusion of 2.6%. The trial exclusion criteria were determined before data collection. The analyses focused on the effect of the outcome of the previous trial; therefore, we also excluded the first trial of the experiment. Note that follow-up tests revealed that results did not change much when we used a stricter cut-off for start RT (2,500 ms instead of 5,000 ms). Furthermore, the RT pattern was very similar when median RTs were analyzed instead of means.

Inferential statistics appear in Table 1. We performed paired *t* tests to contrast the trial types, but we show an overview of the corresponding univariate analyses in Appendix B. For the pairwise comparisons, Hedge’s *g*_av_ is the reported effect size measure (Lakens, 2013).[Table-anchor tbl1]

### Results and Discussion

Start RTs were influenced by the outcome of the previous trial: Subjects started the next trial sooner after a gambled loss (*M* = 485 ms; *SD* = 146) than after a gambled win (*M* = 573 ms; *SD* = 198) or a nongamble (*M* = 669; *SD* = 195); both *p*s < .01 (see Table 1). The difference between trials following a gambled win and trials following a nongamble was also statistically significant, *p* < .01. Thus, compared to a nongambling baseline, gambling on the previous trial generally shortened start RT. Engaging with stimuli that are inconsistently associated with reward (i.e., the gambling option) may temporarily boost dopamine and induce a motivational “approach” state (Robinson, Anselme, Fischer, & Berridge, 2014). Most importantly, this effect was largest after a loss, suggesting that losing points can induce impulsive actions. This finding appears inconsistent with the cognitive control account, which predicts that people should become more cautious (i.e., less impulsive) after a gambled loss than after gambled win or a nongamble. However, it is possible that subjects started the next trial sooner after a loss than after a win because they believed their chances of winning had increased (i.e., the gamblers fallacy). This account is not necessarily inconsistent with the cognitive control account, as it assumes that behavior can be regulated by expectancies about future events; this can happen even when these expectancies or beliefs are incorrect. We explored this idea in Experiment 2.

## Experiments 2a and 2b

Experiment 1 indicates that the outcome of a gamble can influence start RT. In Experiment 2, we further explored the origins of this effect. To examine the motivational consequences of gambling and subjects’ beliefs about upcoming events, on half of the trials we asked them to indicate whether they agreed with the following two statements: “I was pleased with the outcome of the previous trial” and “I think my chances of winning on the next trial have increased” (for a similar procedure, see, e.g., Clark et al., 2013). These statements were presented after the feedback stage (*the previous trial*) but before the new choice options appeared (*the next trial*). To allow a direct comparison with Experiment 1, subjects had to press a key to continue the experiment after each feedback screen.

### Method

#### Subjects

Forty new students (Experiment 2a: *N* = 20; Experiment 2b: *N* = 20; 6 males, age: *M* = 19.6 years, *SD* = 2.0) from the University of Exeter participated for monetary compensation (£5) or partial course credit, plus money won in the gambling task. One subject was replaced in Experiment 2b because they rarely gambled in the task (see Experiment 1).

#### Apparatus, stimuli, procedure, and analysis

These were the same as in Experiment 1 except for the following: In both experiments, responses were registered with a gaming mouse (Razor Deathadder; http://www.razerzone.com/gaming-mice/razer-deathadder). Each trial started with a message “Click to continue.” The trial continued when the subject clicked one of the mouse buttons. On half of the trials (no-rating trials), the gambling task started immediately; on the other half (rating trials), subjects had to rate two statements first. The first statement was always “I was pleased with the outcome of the previous trial (X points)” (X = number of points the subject had won). The second statement was always “I think my chances of winning on the next trial have increased.” The statements appeared in the center of the screen (font: Arial 24 point). Subjects indicated the extent to which they agreed with the statements by clicking with the mouse on a visual analog scale (10 cm) that appeared below the statement. The scale ranged from 0 (*not at all*) to 100 (*very much*). The gambling task started immediately after the second rating response. The order of rating and no-rating trials was fully randomized at the beginning of the experiment (i.e., it was not influenced by the subject’s choices or outcomes of the gambles).

In Experiment 2a, we immediately presented the two choice options together in the gambling task, and subjects could select one of them by clicking with the mouse on the preferred option. A preliminary analysis of this experiment showed that the effect of gambling outcome on choice interacted with the rating manipulation (see Supplementary Materials). To determine whether this was due to the actual rating or to some stimulus-presentation artifact, we ran a second version (Experiment 2b) in which we used the presentation mode of Experiment 1 (i.e., the two options were first presented successively for 1 s, and then they were presented together). Initial analyses showed that Experiment did not interact significantly with the effect of trial outcome. Therefore, we collapsed the data of Experiments 2a and 2b.

For the analyses, we used the same trial exclusion criteria as Experiment 1. This resulted in a data reduction of 11% in Experiment 2a and 4% in Experiment 2b (because the options were immediately presented together in Experiment 2a, choice latencies were longer than the choice latencies in Experiments 1 and 2b). For the ‘Ratings’ ANOVA, generalized eta squared is the reported effect size measure.

### Results and Discussion

#### Start RTs

Subjects executed the start response before the statements were presented; therefore, we collapsed the data of rating and no-rating trials for the start RT analysis. The start RT data were consistent with Experiment 1: subjects started the next trial sooner after a gambled loss (*M* = 416 ms; *SD* = 132) than after a nongamble (*M* = 483 ms; *SD* = 142) or a gambled win (*M* = 455 ms; *SD* = 158); both *p*s < .012 (see Table 1). The difference between trials following a nongamble and trials following a gambled win was marginally significant (two-tailed *p* = .06; one-tailed: *p* = .03).

#### Ratings

The rating for the “pleased with outcome” statement was influenced by the outcome of the previous trial, *F*(2, 78) = 213.5, *p* < .001, η_gen_^2^ = 0.771. Subjects were more pleased with the outcome of the previous trial after a gambled win (*M* = 73, *SD* = 14) than after a nongamble (*M* = 47; *SD* = 14) or a gambled loss (*M* = 16; *SD* = 11); all differences were statistically significant (*p*s < .001).

The rating for the “increased chances of winning” statement was also influenced by the outcome of the previous trial, *F*(2, 78) = 15.9, *p* < .001, η_gen_^2^ = 0.052. Subjects thought that their chances of winning on the next trial had increased more after a gambled win (*M* = 39, *SD* = 23) than after a nongamble (*M* = 35, *SD* = 21) or a gambled loss (*M* = 28, *SD* = 18), which is consistent with the findings of Clark et al. (2013). All differences were statistically significant (*p*s < .015). In other words, we observed a “hot hand” effect (i.e., subjects expected another win after a gambled win more than after a gambled loss; Ayton & Fischer, 2004) rather than a gambler’s fallacy (i.e., the fallacious belief that a run of independent events must be broken). It is possible that the gamblers fallacy influences behavior primarily after longer runs of wins/losses (Ayton & Fischer, 2004); unfortunately, we could not test this idea in Experiment 2 because we did not have enough observations for the various run lengths for each subject.

#### The association between start RT and the ratings

For each subject and statement, we calculated the median rating as a function of the outcome of the previous trial; then we calculated start RT for trials with a rating lower or equal to the corresponding median rating and trials with a rating higher than the corresponding median rating. Six subjects were excluded from these analyses because there were not enough trials for all cells (i.e., *N* < 5).

For both statements, we analyzed start RT with a repeated-measures analysis of variance (ANOVA) with outcome of the previous trial (nongamble, gambled loss, gambled win) and rating (rating ≤ median vs. rating > median) as within-subjects factors. The descriptive statistics appear in Table 2. As discussed above, start RT was influenced by the outcome of the previous trial, but the median split analyses did not reveal other significant effects (see Table B2 in Appendix B).[Table-anchor tbl2]

#### Discussion

Consistent with Experiment 1, we found that start RT was shorter after a gambled loss than after a nongamble (the baseline) or a gambled win. Furthermore, the rating analyses revealed a “hot hand” effect rather than a gamblers fallacy. Thus, the findings of Experiment 2 are inconsistent with the cognitive control accounts discussed in the introduction. According to these accounts, people should become more cautious after a negative or suboptimal outcome. However, our findings indicate that losing led to impulsive actions, rather than cautious actions.

## Experiments 3a and 3b

The results of Experiments 1 and 2 indicate that performance in our gambling task is influenced by the outcome of the previous trial. Work in other domains indicates that sequential effects are often modulated by repetition of information from the previous trial. For example, congruency sequence effects (i.e., a reduction in conflict when conflict also occurred on the preceding trial; Duthoo, Abrahamse, Braem, Boehler, & Notebaert, 2014; Egner, 2008) and other sequential effects (e.g., Henson, Eckstein, Waszak, Frings, & Horner, 2014; Verbruggen, Best, Bowditch, Stevens, & McLaren, 2014) are partly due to the retrieval of stimulus-specific associations from memory (e.g., stimulus-response or stimulus-outcome associations). Even task contexts may become associated with a particular response or outcome (Perruchet, 2015) and influence subsequent performance (e.g., Bouton, 2004; Redish, Jensen, Johnson, & Kurth-Nelson, 2007). Therefore, Experiments 3a and 3b examined whether the sequential effects of winning and losing were also modulated by the repetition of stimulus information.

### Method

#### Subjects

Forty new students (Experiment 3a: *N* = 20; Experiment 3b: *N* = 20; 17 males; age: *M* = 19.4 years, *SD* = 1.3) from the University of Exeter participated for monetary compensation (£5) or partial course credit, plus money won in the gambling task. Three subjects (one in Experiment 3a and two in Experiment 3b) were replaced because they rarely gambled (see Experiment 1).

#### Apparatus, stimuli, procedure, and analyses

These were the same as in Experiment 1 except for the following: The background of the screen (i.e., the wallpaper) was a high-resolution jpeg image of a casino building. There were two images (one of the Bellagio and one of the Venetian—both are casinos in Las Vegas, NV). Image order was randomized; consequently, on approximately half of the trials, the image changed. In both experiments, the image of the casino was presented throughout the whole trial (i.e., from the “Press a key to start the next trial” screen to the “Outcome” screen; see Figure 1). In Experiment 3b, we introduced a short intertrial interval (500 ms), during which a high-resolution image of the famous Las Vegas Strip served as background.

For the analysis, we used the trial exclusion criteria of Experiment 1. This resulted in a data reduction of 3.2% in Experiment 3a and 3.9% in Experiment 3b. The intertrial interval manipulation did not influence performance significantly, so we collapsed the data of Experiments 3a and 3b in the analyses reported below.

### Results and Discussion

Consistent with Experiments 1 and 2, subjects started the next trial sooner after a gambled loss (*M* = 612 ms; *SD* = 204) than after a gambled win (*M* = 723 ms; *SD* = 269) or a nongamble (*M* = 772 ms; *SD* = 197); both *p*s < .001 (see Table 1). The difference between trials following gambled wins and trials following nongambles was also statistically significant (see Table 1). Repetition or alternation of the “casino” background did not significantly influence start RTs (*p*s > .17; Appendix B).^2^ Possibly, subjects did not pay enough attention to the background image, which could have influenced the learning and the retrieval of the relevant associations (e.g., Best, Lawrence, Logan, McLaren, & Verbruggen, 2016). Alternatively, the sequential effects of wins and losses may not be stimulus or context dependent. We will further explore the latter possibility in Experiments 4–5.

## Experiments 4a and 4b

In Experiments 1–3, start RTs were consistently shorter after a gambled loss than after a nongamble, suggesting that losing in our gambling task induces impulsive actions. As indicated in the introduction of Experiments 3a and 3b, many sequential effects are task or context dependent and they do not necessarily transfer from one task to another (Braem, Abrahamse, Duthoo, & Notebaert, 2014). To further explore the generality of the sequential effects of gambling, we used a task-switching manipulation in Experiments 4a and 4b: Subjects alternated between the gambling task in which they could win or lose points and a perceptual decision-making task in which they could not win or lose points.

### Method

#### Subjects

Forty new students (Experiment 4a: *N* = 20; Experiment 4b: *N* = 20; males: 11; age: *M* = 19.8 years, *SD* = 1.5) from the University of Exeter participated for monetary compensation (£5) or partial course credit, plus money won in the gambling task. One subject in Experiment 4a was replaced because they rarely gambled (see Experiment 1).

#### Apparatus, stimuli, and procedure

Subjects continuously alternated between the gambling task and a perceptual decision-making task (in other words, every trial was a task switch; Kiesel et al., 2010; Vandierendonck, Liefooghe, & Verbruggen, 2010). The gambling task was the same as in Experiment 1, apart from the following: every gambling trial started with the message “Press a key to start the next ‘choice’ trial.”

In the perceptual decision-making task, each trial started with the message “Press a key to start the next ‘dark versus light’ trial.” Immediately after subjects had pressed a key, a gray circle (diameter: 5 cm) was presented in the center of the screen against a white background. Subjects had to decide whether it was dark (RGB <127-127-127) or light (RGB >127-127-127), by pressing the left or right arrow key, respectively. They were instructed to respond as quickly and accurately as possible. The circle remained on the screen until a response was executed. Feedback about performance (“Correct response” or “Incorrect response”) was presented for 1 s after every trial.

In Experiment 4a, the difficulty level was continuously adjusted using a two-up/one-down tracking procedure to obtain an error probability of approximately .30 (Leek, 2001). After two correct trials, the RGB difference between dark (e.g., RGB: 120-120-120) and light (e.g., RGB: 134-134-134) was reduced (e.g., RGB: 123-123-123 vs. RGB: 131-131-131), making the discrimination more difficult. The RGB difference increased after each incorrect trial, making the discrimination easier again. To determine whether sequential effects were influenced by overall error rate in the perceptual decision-making task, we made the task slightly easier in Experiment 4b. More specifically, the difficulty level was adjusted using a three-up/one-down tracking procedure to obtain an error probability of approximately .20: The RGB difference was reduced after every three correct trials, but increased after every incorrect trial.

#### Analysis

Subjects continuously alternated between the gambling task and the decision-making task. This influenced our exclusion criteria. In the gambling task, we excluded trials on which start RT was above 5,000 ms or choice RT was above 2,500 ms, and gambling trials on which subjects pressed a key that was not part of the response set (i.e., incorrect gambling trials). We also excluded gambling trials that were preceded by an incorrect gambling trial (i.e., when Trial *n* – 2 was an incorrect gambling trial). We did not exclude gambling trials that were preceded by an incorrect decision-making response (Trial *n* – 1) as there were enough incorrect responses in the perceptual decision-making task for a sequential analysis. In the perceptual decision-making task, we excluded trials that followed an excluded gambling trial (Trial *n* – 1), trials on which start RT was above 5,000 ms, and trials on which response latency was above 2,500 ms. This resulted in a data reduction of 3.8% in Experiment 4a and 4.6% in Experiment 4b. Experiment did not interact with the effects of the outcome of the previous trial, so we collapsed the data of the two experiments in the analyses reported below. For the analysis of choice latencies in the perceptual decision-making task, we included only trials on which subjects responded correctly to the gray circle.

### Results and Discussion

#### Performance in the perceptual decision-making task

Consistent with the gambling experiments, subjects started the perceptual decision-making task sooner after a gambled loss (*M* = 465 ms, *SD* = 161) than after a gambled win (*M* = 575 ms; *SD* = 195) or a nongamble (*M* = 605 ms; *SD* = 185); both *p*s < .01 (see Table 3). The numerical start RT difference between trials that followed a nongamble and trials that followed a gambled win was not statistically significant (see Table 3).[Table-anchor tbl3]

In the perceptual decision-making task, subjects responded faster to the gray circle after a gambled loss (*M* = 903, *SD* = 193) than after a gambled win (*M* = 951 ms, *SD* = 196) or a nongamble (*M* = 939 ms, *SD* = 177); both *p*s < .029 (see Table 3). The choice latency difference between trials that followed a nongamble and trials that followed a gambled win was not statistically significant (see Table 3). Combined, the start RTs and choice latencies indicate that losses can have a “general” effect on impulsive action (i.e., faster responses).

Finally, we analyzed the proportion of correct trials in the perceptual decision-making task. The small numerical accuracy differences between trials following a loss (*M* = .699, *SD* = .076), a gambled win (*M* = .712, *SD* = .107), and a nongamble (*M* = .712, *SD* = .052) were not statistically significant (Table 3 and Appendix B).

#### Performance in the gambling task

Subjects alternated between the gambling task and the perceptual decision-making task. We tested whether the outcome of the previous gambling trial (i.e., Trial *n* – 2) still influenced performance on the current gambling trial (despite the intervening perceptual decision-making trial). For completeness, we also tested whether gambling performance was influenced by the immediately preceding perceptual decision-making trial (i.e., Trial *n* – 1); we present these analyses in Supplementary Materials.

The outcome of a last gambling trial (Trial *n* – 2) still influenced performance on the current gambling trial. Start RT was shorter after a gambled loss (*M* = 524; *SD* = 146) than after a gambled win (*M* = 605; *SD* = 160) or a nongamble (*M* = 572; *SD* = 161); both *p*s < .001 (see Table 1). Thus, doing a nongambling task between two successive gambles did not influence the effect of losses much. However, the intervening task influenced the difference between gambled wins and nongambles. In Experiments 1–3, starts RTs were shorter after a gambled win than after a nongamble. In this experiment, start RT was longer when Trial *n* – 2 was a gambled win compared with a nongamble (see Table 1).

## Experiment 5

Experiments 4a and 4b showed that losing a gamble can influence performance in a seemingly unrelated perceptual decision-making task. In other words, losses generally induce faster responses on subsequent trials (whatever the nature of that trial). Furthermore, we found that performing a difficult discrimination task did not modulate the effect of losses on start RT in the gambling task much. These findings are remarkable, and could indicate that cognitive control processes have little influence on loss-induced impulsivity. Therefore, we explored in Experiment 5 whether the effect of losses on response latencies was also observed in a task in which subjects typically respond with caution. More specifically, subjects alternated between the gambling task and a stop-signal task (Verbruggen & Logan, 2008). As discussed in the introduction, many studies have shown that people slow down and respond more cautiously when they expect a stop signal to occur. Therefore, Experiment 5 explored whether a loss in the gambling task could influence response latencies in the stop-signal task as well.

### Method

#### Subjects

Forty new students (11 males; age: *M* = 19.9 years, *SD* = 2.5) from the University of Exeter participated for monetary compensation (£5) or partial course credit, plus money won in the gambling task. One subject was replaced because they rarely gambled (see Experiment 1), and two subjects were replaced because *p*_correct_ in the stop-signal task was below .80.

#### Apparatus, stimuli, and procedure

Subjects continuously alternated between the gambling task and a stop-signal task (in other words, every trial was a task switch). The gambling task was the same as in Experiment 4.

In the stop-signal task, each trial started with the message “Press a key to start the next ‘dark vs. light’ trial”. Immediately after subjects had pressed a key, a gray circle (diameter: 5 cm) was presented in the center of the screen against a white background. Subjects had to decide whether it was dark (RGB: 88-88-88) or light (RGB: 167-167-167) by pressing the left or right arrow key, respectively. The circle remained on the screen for 1,500 ms, regardless of RT. On 25% of the trials (stop-signal trials), the circle turned blue (RGB: 0-150-255) after a variable delay, instructing the subjects to withhold their response. The stop-signal delay was initially set at 500 ms, and continuously adjusted according to a one-up/one-down tracking procedure to obtain a probability of stopping of .50: stop-signal delay decreased by 50 ms when a subject responded on a stop-signal trial, but increased by 50 ms when they successfully stopped (Verbruggen & Logan, 2009a).

#### Analyses

For the gambling task, we used the same exclusion criteria as in Experiment 4. In the stop-signal task, we excluded trials that followed an excluded gambling trial and trials on which start RT was above 5,000 ms, which resulted in a data reduction of 2.5%. For the analysis of go latencies in the stop-signal task, we included only trials on which subjects responded correctly to the gray circle. There were not enough stop-signal trials to examine the effect of the gambling outcome on stop performance.^3^

### Results and Discussion

#### Go performance in the stop-signal task

Subjects started the stop-signal task sooner after a gambled loss (*M* = 633 ms, *SD* = 266) than after a gambled win (*M* = 742, *SD* = 318) or a nongamble (*M* = 751 ms, *SD* = 254); both *p*s < .01 (see Table 3). The numerical difference between trials that followed a nongamble and trials that followed a gambled win was not statistically significant (see Table 3).

Go accuracy was lower after a gambled loss (*M* = .905, *SD* = .073) than after a nongamble (*M* = .926, *SD* = .055) or a gambled win (*M* = .932, *SD* = .080); both *p*s < .032 (see Table 3). Go latencies (i.e., once the stop-signal task had been initiated) were similar for trials following a loss (*M* = 725 ms, *SD* = 176), a nongamble (*M* = 737, *SD* = 168), and a gambled win (*M* = 723, *SD* = 177); none of the numerical differences were statistically significant (see Table 3). This finding suggests that proactive control adjustments can partly counteract loss-induced impulsivity. The absence of a go latency difference indicates the go accuracy difference is not due to a simple speed/accuracy trade-off in the stop task itself. However, it is possible that by starting the next trial sooner after a loss, subjects were less prepared, resulting in lower accuracy.

#### Performance in the gambling task

We explored whether the outcome of the last gambling trial (Trial *n* – 2) still influenced performance on the current gambling trial (trial *n*), despite the intervening stop-signal task (Trial *n* – 1). Start RT was 720 ms (*SD* = 247) after a gambled loss, 736 ms (*SD* = 240) after a gambled win, and 748 ms (*SD* = 242) after a nongamble. However, the individual contrasts were no longer significant (see Table 1). Thus, performing a cognitive control tasks in which people are usually cautious attenuated loss-induced impulsivity.

## Combined Analysis Start Latencies

The results of Experiments 1–5 indicate that gambled losses and wins affect start RTs on the next trial. Before we discuss the implications of these findings, we report the outcome of a two extra analyses.

**Figure 2 fig2:**
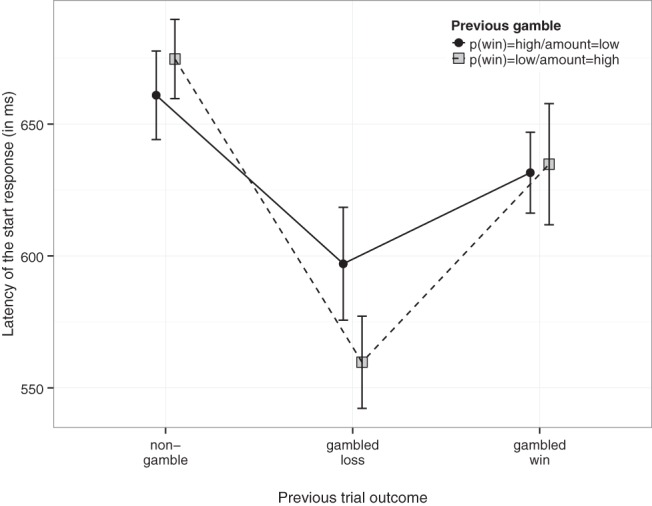
Start reaction time as a function of the outcome of the previous trial and *p*_win_ and amount of the previous gamble (When probability of winning was low, the amount was always high; see Appendix A). The error bars reflect within-subject confidence intervals (Morey, 2008). Note that we observed a similar numerical pattern when all subjects were included; however, we could not perform univariate analyses when all subjects were included because of missing cells.

First, we explored whether the effect of gambled wins and losses was influenced by the probability of winning and the amount of the previous gamble. As discussed in the Method section of Experiment 1, the amount subjects could win when they gambled increased when probability of winning decreased (and vice versa; see Appendix A). To obtain sufficient observations for this analyses (*N* ≥ 5 for each cell), we collapsed trials for which *p*_win_ of the previous gamble option was .67 and .50 (that is, a relatively “high” *p*_win_ but lower amount), and trials for which *p*_win_ of the previous gamble option was .25 or .33 (that is, lower *p*_win_ but higher amount). The corresponding amounts are shown in Appendix A. Because some subjects only gambled when *p*_win_ was either high or low, we had to exclude 43 subjects (leaving 137 subjects for the analyses reported below). Start RT was analyzed by means of a 3 (Outcome Previous Trial: nongamble, gambled loss, gambled win) by 2 (*p*_win_ of the Gamble Option of the Previous Trial: high or low) repeated-measures ANOVA. Figure 2 shows that the effect of a gambled loss on start RT of the next trial was more pronounced when the probability of winning the gamble was low (and therefore the potential win was a large amount). The interaction between trial outcome and probability of winning was significant, *F*(2, 272) = 5.457, *p* = .005, η_gen_^2^ = 0.002. A follow-up *t* test revealed that start RT after a gambled loss was shorter when the gamble was associated with a low *p*_win_ but high amount, compared to when it was associated with a higher *p*_win_ but a lower amount; *t*(136) = 2.91, *p* = .004, *g*_av_ = .164, Bayes factor (*BF*) = 5.35. Start RTs after a nongamble or a gambled win were not modulated much by *p*_win_ of the previous trial (both *p*s > .148; furthermore, Bayesian analyses provided substantial support for the null hypothesis, *BF*s < 0.27). We discuss the implications of this finding in the General Discussion. Note that in this combined analysis, the main effect of trial outcome is similar to the effects observed in Experiments 1–5; thus, the exclusion of 43 subjects did not alter the overall data pattern much.[Fig-anchor fig2]

Second, we analyzed the difference between start RT of the current trial (Trial *n*) and start RT of the immediately preceding trial (Trial *n* – 1). Measurements of posterror slowing can be contaminated by global fluctuations in performance over the course of an experiment. For example, when subjects are temporarily distracted, they are more likely to make an error and average RT is likely to be higher. However, this will influence the measurement of posterror slowing because “posterror trials” are more likely to come from blocks in which the subject was distracted (hence, RT was long) than from blocks in which the subject was focused (hence, RT was short). There is a solution for this problem: posterror slowing can be quantified as the RT difference between the posterror trial and the associated preerror trial (e.g., Dutilh, van Ravenzwaaij, et al., 2012). Similar solutions have been proposed to control for global fluctuations in other paradigms (e.g., Nelson, Boucher, Logan, Palmeri, & Schall, 2010).

It is possible that our start RT measurements were also contaminated by global fluctuations (e.g., if subjects only gambled when they were focused or motivated, start RT should be shorter after a gamble than after a nongamble). To control for such global effects, we analyzed the “Trial *n* minus Trial *n* – 1 difference.” For this analysis, we excluded all trials that were excluded in the main analyses, plus trials for which the absolute start RT difference with the previous trial was larger than 5 s (we included them in the main analyses above to boost the trial numbers). The difference score analysis showed that the start RT difference was negative (indicating shorter latencies) after a gambled loss (*M* = −72 ms, 95% confidence interval [CI] [−85, −58]), close to zero after a gambled win (*M* = 2 ms, 95% CI [−12, 16]), and positive after a nongamble (*M* = 16 ms, 95% CI [9, 24]). The “gambled loss versus gambled win” and the “gambled loss versus nongamble” differences were both statistically significant; *t*(179) = 6.74, *p* < .001, *g*_av_ = .78, *BF* = 3.18 × 10^7^, and *t*(179) = −9.23, *p* < .001, *g*_av_ = 1.21, *BF* = 7.12 × 10^13^, respectively. Thus, the sequential effect of losses on start RTs is not due to global fluctuations. The “gambled win versus nongamble” difference was not significant, *t*(179) = 1.54, *p* = .13, *g*_av_ = .19, *BF* = 0.27.

## Overview of the Choice Analyses

For completeness, we also analyzed how the probability of gambling and the latency of the choice response were influenced by the outcome of the previous trial. In Appendices C and D, we present the descriptive and inferential statistics for each individual experiment. In this section, we will focus on the combined analysis only.

### Probability of Gambling in Experiments 1–5

We combined the *p*_gamble_ data of all experiments to establish whether the postreinforcement effect (decreased gambling after a win) and, loss chasing (increased gambling after a loss), or a combination of the two influenced the probability of gambling. Note that we excluded the rating trials of Experiment 2 from this combined analysis because analyses had revealed that the ratings influenced subsequent performance (see Appendix C).

*p*_gamble_ was lowest after a gambled win (*M* = .44, *SD* = .20), intermediate after a nongamble (*M* = .48, *SD* = .20), and highest after a gambled loss (*M* = .51, *SD* = .19). All differences were statistically significant (see Table 4). In sum, both postreinforcement effects and loss chasing influenced choice in our gambling task (although the effect sizes reported in Table 4 and Appendix C indicate that the loss chasing effect was relatively small; furthermore, the Bayesian analysis of the “nongamble vs. gambled loss” difference only provided anecdotal support for the alternative hypothesis).[Table-anchor tbl4]

### Choice RT in Experiments 1–5

After both options (i.e., the nongamble option and the gamble option) had been presented separately, subjects saw them again together (one on the left and one on the right of the screen). At this point, they could choose one option by pressing the left- or right-arrow key of the keyboard for the left or right option, respectively (see Figure 1). Here we examined whether the latency of the choice responses (choice RTs) were influenced by the outcome of the previous trial.

Choice RT was shortest after a gambled loss (*M* = 698 ms, *SD* = 215 ms), intermediate after a nongamble (*M* = 713 ms, *SD* = 227), and longest after a gambled win (*M* = 731 ms, *SD* = 239). All individual differences were statistically significant (see Table 4). The difference between gambled losses and nongambles is consistent with the start RT differences reported in the main manuscript. However, choice RT was longer after a gambled win than after a nongamble, whereas start RT was *shorter* after a gambled win than after a nongamble.

## General Discussion

The present study explored the effects of winning and losing on impulsive action. Work in various domains indicates that people slow down after errors, conflict, or suboptimal outcomes. In other words, they become more cautious. Such sequential effects have been attributed to changes in cognitive control settings. The cognitive control account predicted that response latencies should increase after a gambled loss compared with a nongambling baseline or a gambled win in our gambling task. However, in all experiments, start RT was shorter after a gambled loss than after a gambled win or a nongamble. These findings are inconsistent with the idea that people become more cautious after a loss; instead, they indicate that losses in a gambling task can induce impulsive actions.^4^ Furthermore, the results of Experiment 4 and the similar (albeit smaller) effects on choice RTs in the gambling task (see Overview of the Choice Analyses) indicate that loss-induced impulsivity does not only influence task-unspecific responses (i.e., the start response, for which there is no correct or incorrect response) but also task-specific responses (i.e., the choice response in the perceptual-decision-making task and the choice response in the gambling task). In other words, losses seem to have a general effect on actions.

In Experiments 1 and 3, start RT was also significantly shorter after a gambled win than after a nongamble (similar numerical trends were observed in Experiment 2 and in the neutral tasks of Experiments 4 and 5). This suggests that start RT is generally faster after a previous gamble than after a nongamble, although it should be noted that the overall numerical difference between nongambles and gambled wins was not significant when we controlled for global fluctuations (see above).

### Affective Consequences of Gambling and Negative Outcomes

Our findings are inconsistent with popular cognitive control accounts; instead, they suggest emotional influences on action control. Affect has multiple dimensions or components that can be influenced by the outcome of a gamble, including affective valence and motivational intensity.

Ratings obtained in Experiment 2 suggest that a gambled win induces positive affect, whereas a gambled loss induces negative affect. Negative affect after a loss could reflect frustration, regret, or disappointment. Frustration refers to a strong negative affective state that is induced by a failure to obtain an incentive or the blockage of a desired goal. Disappointment refers to the realization that the outcome is worse than expected or hoped for (e.g., the amount associated with the gamble option is 60 points, but the outcome is 0 points), whereas regret^5^ refers to the realization that another choice (i.e., not gambling in our task) would have produced a better outcome (e.g., 30 points). Such negative affective states can modulate motivational intensity. Previous work suggests that subsequent behavior is energized (Amsel, 1958, 1992; Carver, 2006; Carver & Harmon-Jones, 2009; Mikulincer, 1988) and approach tendencies are intensified (Harmon-Jones, Harmon-Jones, & Price, 2013) in an attempt to overcome frustration. For example, Amsel (1958) placed hungry rats in a box that consisted of two runways in which the rats expected to find food. He observed that rats ran faster in the second runway when they did not find the expected food in the first runway. He labeled this the “frustration effect” (Amsel, 1958, 1992). Similarly, a recent study showed that rats consumed their food faster after they had experienced “regret” compared with a control condition (Steiner & Redish, 2014).

The start RT results of the present study are consistent with the idea that negative outcomes can increase motivational intensity and approach behavior. In our task, each trial started with the presentation of a certain amount of points, and subjects could lose these points when they gambled. In other words, a gambled loss (i.e., outcome = 0 points) in our task is a negative event. Therefore, the shorter start RTs (and choice RTs) after a loss compared with the nongambling baseline indicate that negative affect can induce faster responses (i.e., impulsive actions). Note that findings of the combined analyses (see Figure 2) suggests that a failure to obtain a large reward (i.e., when *p*_win_ was low) had a stronger effect on start RT than a failure to obtain a smaller reward (i.e., when *p*_win_ was high). This suggests that start RT was modulated by frustration or the disappointment of not obtaining the larger amount of the gamble.

Many theories assume that affective states facilitate actions and induce impulsivity (e.g., Cyders & Smith, 2008; Frijda, Ridderinkhof, & Rietveld, 2014). Positive affect is typically associated with approach behavior, whereas negative affect is typically associated with avoidance behavior (Braver et al., 2014). Thus, increased impulsivity or approach behavior after negative events may seem counterintuitive. However, it could be functionally relevant. Quickly continuing the game after a gambled loss may help to escape or relieve the negative affective state (see also e.g., Billieux et al., 2010; Cyders & Smith, 2008). It may also help to close the gap between the current state (no reward) and the desired state (a reward; cf. Braver et al., 2014).

Work in other control domains also suggests that emotional and motivational influences can modulate sequential effects (although not necessarily in the same way as in the present study). For example, in Stroop and flanker tasks, conflict resolution and between-trial adaptations are enhanced when an “avoidance” response or state is induced (Schouppe, De Houwer, Ridderinkhof, & Notebaert, 2012; Hengstler, Holland, van Steenbergen, & van Knippenberg, 2014). By contrast, conflict adaptations are reduced when reward is delivered (e.g., van Steenbergen, Band, & Hommel, 2009). More generally, some have argued that conflict or errors could be construed as aversive events that are to be avoided in the future (Botvinick, 2007; Dreisbach & Fischer, 2012; Fritz & Dreisbach, 2013). This aversive signal could then lead to adjustments in behavior. Thus, motivation and emotion can influence sequential control adjustments in conflict tasks. This conclusion is generally consistent with the findings of the present study. However, in the case of conflict and errors, these adjustments lead to response slowing (i.e., avoidance behavior), whereas we observed increased impulsivity (i.e., approach behavior) after a negative outcome. In other words, the nature of the affect plays a critical role.

### Probability of Gambling

The combined analysis revealed that *p*_gamble_ was higher after a gambled loss than after a nongamble, and lowest after a gambled win. Again, this pattern is inconsistent with the idea that people become more cautious after a loss. However, our *p*_gamble_ results are consistent with previous studies (see the introduction), and support the idea that subjective value or utility of options is context dependent and highly malleable (Stewart, Reimers, & Harris, 2015; Vlaev, Chater, Stewart, & Brown, 2011). For example, several models of decision-making assume that the subjective value of an option depends on immediately preceding gains and losses (for a short review, see Smith et al., 2009). Reduced gambling after a gambled win (especially after a large win) and increased gambling after a loss are consistent with this idea.

The *p*_gamble_ results of the present study are also consistent with the idea that affective components can modulate decision-making. Emotion-based theories of decision making propose that emotional states and feelings about expected outcomes influence choice (for reviews, see Coricelli, Dolan, & Sirigu, 2007; Connolly & Zeelenberg, 2002; Mellers & McGraw, 2001). Consistent with this idea, Campbell-Meiklejohn, Woolrich, Passingham, and Rogers (2008) found in a functional MRI study that decisions to quit gambling were associated with increased activation of brain regions associated with the anticipation of negative events. By contrast, loss chasing was associated with activation of brain regions often linked to reward processing, appetitive states, and the experience of urges and cravings (Campbell-Meiklejohn et al., 2011; Campbell-Meiklejohn et al., 2008). Emotion-based theories of decision making could explain why people gamble less after a gambled win than after a nongamble in our task: After a gambled win, people are in a positive affective state, and the anticipatory disappointment and regret associated with a loss may steer them away from gambling again. Positive feelings may also induce “coasting” (Carver, 2003), reducing further gambling efforts. By contrast, engaging in a gamble after a loss may reduce the aversive consequences of the loss.

Thus, wins and losses influenced both impulsive action and choice in our task. But were these effects on action and choice related? An extra analysis revealed that the “gambled loss versus gambled win” start RT difference did not correlate with the “gambled loss versus gambled win” *p*_gamble_ difference, *r*(179) = −.05, *p* = .523. This (partial) dissociation between impulsive action (start RT) and choice (*p*_gamble_) suggests that outcomes of a gamble can have distinct effects on motivational intensity and affective valence. More generally, it is consistent with the idea that expression of behavior involves two components, namely what to do and how vigorously to do it (Guitart-Masip, Beierholm, Dolan, Duzel, & Dayan, 2011; see also Berridge, Robinson, & Aldridge, 2009). In many situations, these components are tightly coupled, but clinical, cognitive and neuroscience research indicates that they can be dissociated (Berridge et al., 2009). The results of the present study may be another example of such a dissociation.

Finally, the analyses of the individual experiments (see Appendix C) revealed another interesting finding. Experiment 2 suggests that encouraging subjects to pause and reflect influenced their subsequent gambling behavior: compared with the nongambling baseline, *p*_gamble_ decreased after a loss on rating trials; by contrast, there was a small (numerical) increase on no-rating trials (see Appendix C). This is consistent with previous studies that showed that presenting feedback and encouraging subjects to reflect on their choices influences subsequent decision-making (Brand, 2008; Corr & Thompson, 2014). The findings of Experiment 4 further show that simply interrupting the flow of the gambling task is not sufficient. In this experiment, the outcome of a gamble influenced choice on the next gambling trial even when subjects had to perform an unrelated task between the two successive gambles. In other words, unless people are encouraged to reflect, interrupting the game does not seem to modulate the effect of trial outcome on *p*_gamble_ much. However, the additional rating analyses for Experiment 2 suggest that the beneficial effect of reflecting on the outcome may be counteracted by fallacious beliefs, as *p*_gamble_ increased after a loss when subjects thought that their chances of winning had increased (see Appendix C). This finding could be important for clinical applications, as it suggests that merely introducing a “pause for reflection” may not be sufficient for reducing possible harmful effects of gambling in people with gambling-related problems unless cognitive biases are also corrected.

### Alternative Explanations

We attributed shorter start RTs after a gambled loss to the affective consequences of gambling. Based on our findings, we can rule out several alternative explanations. First, we can rule out that the start RT pattern was due to expectancy violations. Previous work indicates that unexpected events can slow responding (e.g., Leiva, Parmentier, Elchlepp, & Verbruggen, 2015; Notebaert et al., 2009). When *p*_win_ of the gamble option was .25, subjects could expect a loss when they selected the gamble (and a gambled win was unexpected); by contrast, when *p*_win_ of the gamble option was .67, subjects could expect a win when they selected the gamble (and a loss was unexpected). Figure 2 shows that *p*_win_ and the associated amount of the gamble influenced the start RT after a gambled loss but not after a gambled win. Furthermore, start RTs were longest after the most predictable outcome (i.e., the nongamble). Consequently, it seems highly unlikely that the overall start RT pattern (start RT gambled loss < start RT gambled win < start RT nongamble) was due to expectancy violations.

Second, we can rule out the possibility that the start RT pattern was due to differences in feedback complexity. Processing complex feedback may slow responding on subsequent trials. However, the difference between “low *p*_win_/high amount” losses and “high *p*_win_/low amount” losses (see Figure 2) is inconsistent with a feedback-complexity account. After all, the exact same feedback message was presented on both trial types (i.e., “outcome = 0 points”). Furthermore, we propose that processing the feedback was most straightforward on nongambling trials because subjects already knew the outcome of the trial before the feedback was presented. Finally, in an extra analysis (not shown), we excluded trials that followed a trial on which the guaranteed amount associated with the nongambling option was 30 or 40. As shown in Appendix A, these amounts were occasionally awarded on gamble trials as well. Thus, presenting these amounts after a nongamble could have induced some conflict (assuming that these amounts were associated with gambling). However, the extra analyses revealed that start RT was still shorter after a loss than after a nongamble (*p* < .001) or a gambled win (*p* < .001) when these trials were excluded. Thus, we can rule out a feedback-complexity account.

Third, we used nongambling trials as a baseline to determine if gambled losses induced impulsive actions or if gambled wins induced cautious actions. But subjects still received some points when they did not gamble. In other words, it could be argued that the nongambled trials were similar to gambled wins. Consequently, the start RT pattern could reflect a postreinforcement pause (i.e., slower responding after the delivery of a reward), rather than a loss-induced impulsivity effect. However, the ratings in Experiment 2 indicate that nongambles were treated differently to gambled wins. Furthermore, the postreinforcement pause account predicts that start latencies should increase as a function of the magnitude of the reward (Dixon et al., 2013). By contrast, we found that start latencies were longer after a nongamble (lower amounts) than after a gambled win (higher amounts). Finally, the results depicted in Figure 2 suggest that the start RT pattern is largely due to losses encouraging faster responses (rather than wins encouraging slower responses).

### Negative Urgency

**Figure 3 fig3:**
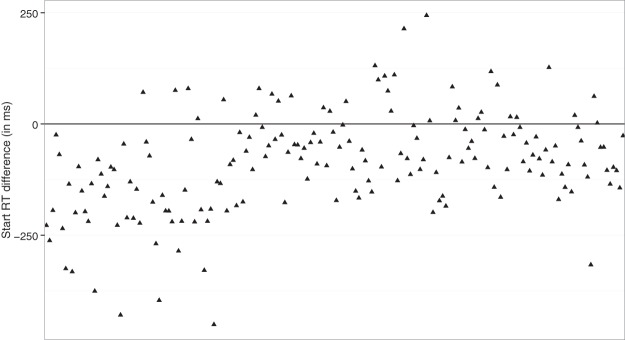
The start reaction time difference between trials following a gambled loss and trials following a nongambling (i.e., the baseline) for each individual (total *N* = 180; the *x*-axis shows “experimental subject”). Negative values indicate loss-induced impulsivity.

The present study focused on “state” impulsivity (i.e., transient changes in action control). However, Figure 3 shows that there were large individual differences. How people respond to a loss could be influenced by various personality traits. Trait impulsivity is a multifaceted construct. Several studies indicate that acting impulsively in response to negative events (“negative urgency” trait) can be dissociated from other impulsivity traits (Cyders & Smith, 2008). Importantly, negative urgency or mood-based rash action can be associated with various behavioral addictions (including gambling; Billieux et al., 2012; Michalczuk, Bowden-Jones, Verdejo-Garcia, & Clark, 2011) substance-use disorders, and risk taking (Settles et al., 2012). Thus, how people respond to emotional events is clinically relevant.[Fig-anchor fig3]

We propose that our task measured negative urgency-like states. It could be interesting to link state- and trait impulsivity. For example, Gipson et al. (2012) found that subjects scoring high in negative urgency showed increased response vigor (indicated by the number of mouse clicks executed to obtain a new reward) and increased frustration following unexpected reward omission compared to subjects low in negative urgency. In the present study, we did not include self-report questionnaires. Therefore, a future goal of our research program is to further explore individual differences in our paradigm, and examine how these are related to personality traits such as negative urgency.

## Conclusions

Research on gambling challenges many psychological and economic models of decision making and human behavior (Clark, Studer, Bruss, Tranel, & Bechara, 2014). In these models, humans are often portrayed as rational beings who learn from their mistakes and optimize their behavior. In the present study, we explored how people adjust behavior after they have lost a gamble. We found that that gambled losses induced impulsive actions. Furthermore, we observed reduced risk-taking after a win but increased risk-taking after a loss. In sum, we found only limited support for the idea that people increase control settings after a negative outcome in gambling tasks. Instead, our results indicate that emotional and motivational factors largely determine how people respond to losses in a gambling task.

## Supplementary Material

10.1037/xhp0000284.supp

## Figures and Tables

**Table 1 tbl1:** Overview of Planned Comparisons to Explore the Effect of the Previous Gamble on the Start RT of the Gambling Task in Experiments 1–5

Experiment	diff	Lower CI	Upper CI	*t*	*p*	*g*_av_	*BF*
Experiment 1							
Nongamble vs. gambled loss	184	141	227	8.905	<.001	1.057	4.15 × 10^5^
Nongamble vs. gambled win	96	48	145	4.181	.001	.482	66.17
Gambled loss vs. gambled win	−87	−132	−42	−4.080	.001	.498	54.10
Experiment 2							
Nongamble vs. gambled loss	67	42	92	5.376	<.001	.482	4,913
Nongamble vs. gambled win	28	−1	57	1.936	.060	.184	.92
Gambled loss vs. gambled win	−39	−68	−9	−2.662	.011	.265	3.69
Experiment 3							
Nongamble vs. gambled loss	160	121	199	8.321	<.001	.895	3.04 × 10^7^
Nongamble vs. gambled win	49	1	97	2.080	.044	.231	1.18
Gambled loss vs. gambled win	−111	−170	−52	−3.819	<.001	.529	60.15
Experiment 4							
Nongamble vs. gambled loss	48	26	69	4.453	<.001	.306	340.50
Nongamble vs. gambled win	−33	−56	−10	−2.952	.005	.205	7.02
Gambled loss vs. gambled win	−81	−111	−51	−5.517	<.001	.522	7,459
Experiment 5							
Nongamble vs. gambled loss	28	−7	62	1.616	.114	.112	.56
Nongamble vs. gambled win	12	−14	37	.938	.354	.048	.26
Gambled loss vs. gambled win	−16	−50	19	−.930	.358	.065	.26
*Note.* RT = reaction time; diff = difference; CI = confidence interval; *g*_av_ = Hedge’s average *g*; *BF* = Bayes factor, which is an odds ratio: It is the probability of the data under one hypothesis relative to that under another. Evidence categories for Bayes Factor: *BF* < .33 = substantial evidence for Hypothesis (H)_0_; 1/3–1 = anecdotal evidence for H_0_; 1 = no evidence; 1–3 = anecdotal evidence for H_A_; 3–10 = substantial evidence for H_A_; *BF* > 10 = strong to decisive evidence for H_A_. H_0_ = no difference between the trial types; H_A_ = a difference between the trial types. We calculated the Bayes factors with the BayesFactor package in R, using the default prior (.707). Experiment 1, *df* = 19; Experiments 2–5, *df* = 39.							

**Table 2 tbl2:** Overview of the Ratings and Mean Start RT as a Function of the Median Split (Rating ≤ Median Rating or > Median Rating) and the Preceding Gambling Trial in Experiment 2

	Non-gamble		Loss		Gambled win	
Statement	≤Median	>Median	≤Median	>Median	≤Median	>Median
Pleased with the outcome						
Rating	37 (16)	57 (12)	10 (8)	26 (15)	58 (16)	83 (12)
Start RT	487 (168)	505 (181)	397 (132)	415 (136)	456 (200)	443 (148)
Increased chances of winning						
Rating	29 (17)	46 (19)	20 (16)	40 (19)	34 (20)	53 (23)
Start RT	483 (175)	506 (171)	395 (130)	420 (138)	450 (176)	444 (173)
*Note.* RT = reaction time. *SD* in parentheses.						

**Table 3 tbl3:** Overview of Planned Comparisons to Further Explore the Effect of the Previous Gamble on Performance in the Perceptual Decision-Making Task of Experiment 4 and the Go Task of Experiment 5

Experiment	diff	Lower CI	Upper CI	*t*	*p*	*g*_av_	*BF*
Experiment 4							
Start RT							
Nongamble vs. gambled loss	140	111	168	9.872	<.001	.798	2.38 × 10^9^
Nongamble vs. gambled win	30	−11	71	1.485	.146	.156	.47
Gambled loss vs. gambled win	−110	−138	−81	−7.805	<.001	.611	6.77 × 10^6^
Go accuracy							
Nongamble vs. gambled loss	.013	−.011	.037	1.102	.277	.201	.30
Nongamble vs. gambled win	.000	−.034	.034	.022	.983	.005	.17
Gambled loss vs. gambled win	−.013	−.047	.022	−.746	.460	.137	.22
Go RT							
Nongamble vs. gambled loss	35	4	67	2.284	.028	.190	1.72
Nongamble vs. gambled win	−13	−37	12	−1.043	.303	.067	.28
Gambled loss vs. gambled win	−48	−76	−21	−3.525	.001	.245	28.07
Experiment 5							
Start RT							
Nongamble vs. gambled loss	118	75	161	5.544	<.001	.450	8073
Nongamble vs. gambled win	9	−31	49	.456	.651	.032	.19
Gambled loss vs. gambled win	−109	−147	−72	−5.900	<.001	.370	23,318
Go accuracy							
Nongamble vs. gambled loss	.021	.002	.039	2.240	.031	.318	1.58
Nongamble vs. gambled win	−.006	−.026	.014	−.611	.545	.089	.20
Gambled loss vs. gambled win	−.027	−.046	−.007	−2.744	.009	.346	4.41
Go RT							
Nongamble vs. gambled loss	12	−5	30	1.423	.163	.070	.432
Nongamble vs. gambled win	14	−3	31	1.711	.095	.081	.646
Gambled loss vs. gambled win	2	−14	17	.247	.806	.011	.176
*Note.* RT = reaction time; diff = difference; CI = confidence interval; *g*_av_ = Hedge’s average *g*; BF = Bayes factor. For all comparisons, *df* = 39.							

**Table 4 tbl4:** Overview of the Combined Analysis to Explore the Effect of the Previous Gamble on Choice Data

Dependent variable	diff	Lower CI	Upper CI	*t*	*p*	*g*_av_	*BF*
*p*_gamble_							
Nongamble vs. gambled loss	−.029	−.052	−.005	2.434	.016	.149	1.47
Nongamble vs. gambled win	.044	.067	.067	3.725	<.001	.217	60.12
Gambled loss vs. gambled win	.073	.049	.096	6.149	<.001	.370	6.77 × 10^6^
Choice latency							
Nongamble vs. gambled loss	14	3	26	2.499	.013	.065	1.71
Nongamble vs. gambled win	−18	−32	−4	2.486	.014	.078	1.66
Gambled loss vs. gambled win	−32	−45	−20	4.950	<.001	.143	6,833
*Note.* diff = difference; CI = confidence interval; *g*_av_ = Hedge’s average *g*; BF = Bayes factor.							

**Table B1 tbl5:** Overview of Univariate Analyses to Explore the Effect of the Previous Gamble on the Start RT of the Current Gambling Trial in Experiments 1–5

Experiment	*df*1	*df*2	Sum of squares effect	Sum of squares error	*F*	*p*	η_gen_^2^
Experiment 1	2	38	337,735	179,253	35.798	<.001	.153
Experiment 2	2	78	90,207	299,992	11.727	<.001	.036
Experiment 3							
Outcome Trial *n*–1	2	78	1,080,000	1,848,854	22.800	<.001	.083
Casino alternation (CA)	1	39	63,800	1,298,828	1.910	.174	.005
Outcome by CA	2	78	18	1,212,027	.001	.999	.000
Experiment 4	2	78	132,183	237,340	21.720	<.001	.044
Experiment 5	2	78	15,412	385,935	1.557	.217	.002
*Note.* In Experiment 3, we also included Casino Alternation as a within-subjects factor. RT = reaction time; gen = generalized.							

**Table B2 tbl6:** Overview of Univariate Analyses to Explore the Effect of the Median Split (Rating: rating ≤ median vs. rating > median) and the Preceding Gambling Trial (Outcome: nongamble, gambled loss, gambled win) on Start RT of the Current Gambling Trial in Experiment 2

Statements	*df*1	*df*2	Sum of squares effect	Sum of squares error	*F*	*p*	η_gen_^2^
“Pleased with outcome”							
Outcome Trial *n* – 1	2	66	275,878	746,990	12.188	<.001	.050
Rating split	1	33	2,917	304,939	.316	.578	.001
Outcome:rating split	2	66	11,304	367,909	1.014	.368	.002
“Increased chances of winning”							
Outcome Trial *n* – 1	2	66	259,152	725,899	11.781	<.001	.048
Rating split	1	33	9,990	255,436	1.291	.264	.002
Outcome:rating split	2	66	11,494	369,234	1.027	.364	.002
*Note.* gen = generalized.							

**Table B3 tbl7:** Overview of Univariate Analyses to Explore the Effect of the Previous Gamble on Performance in the Perceptual Decision-Making Task of Experiment 4 and the Go Task in Experiment 5

Variables	*df*1	*df*2	Sum of squares effect	Sum of squares error	*F*	*p*	η_gen_^2^
Experiment 4							
Start RT	2	78	433,299	417,553	40.471	<.001	.101
Go accuracy	2	78	.004	.369	.467	.629	.006
Go RT	2	78	49,764	298,442	6.503	.002	.012
Experiment 5							
Start RT	2	78	346,073	620,776	21.742	<.001	.036
Go accuracy	2	78	.016	.144	4.233	.018	.027
Go RT	2	78	4,681	104,288	1.751	.180	.001
*Note.* gen = generalized; RT = reaction time.							

**Table C1 tbl8:** Overview of Probability of Gambling for Experiments 1–5 as a Function of the Outcome of the Last Gambling Trial

	Nongamble		Loss		Gambled win	
Experiment	*M*	*SD*	*M*	*SD*	*M*	*SD*
Experiment 1	.517	.159	.529	.166	.452	.188
Experiment 2						
No rating	.511	.213	.517	.210	.444	.240
Rating	.518	.226	.457	.212	.457	.231
Experiment 3						
Casino repetition	.443	.221	.505	.190	.409	.222
Casino alternation	.439	.227	.505	.194	.427	.221
Experiment 4	.458	.204	.483	.186	.412	.192
Experiment 5	.492	.178	.521	.186	.462	.191
*Note.* In Experiment 2, we distinguished between rating and no-rating trials. In Experiment 3, we distinguished between casino-repetition and casino-alternation trials. In Experiments 4 and 5, subjects performed a nongambling task between two successive gambling trials.						

**Table C2 tbl9:** Overview of Planned Comparisons to Explore the Effect of the Previous Gamble on Probability of Gambling in Experiments 1–5

Experiment	diff	Lower CI	Upper CI	*t*	*p*	*g*_av_	*BF*
Experiment 1							
Nongamble vs. gambled loss	−.011	−.084	.061	−.325	.749	.068	.24
Nongamble vs. gambled win	.065	.003	.128	2.187	.041	.368	1.61
Gambled loss vs. gambled win	.076	.013	.14	2.52	.021	.423	2.79
Experiment 2: no rating							
Nongamble vs. gambled loss	−.006	−.066	.054	−.210	.835	.029	.17
Nongamble vs. gambled win	.067	.004	.129	2.168	.036	.292	1.39
Gambled loss vs. gambled win	.073	.012	.134	2.418	.020	.322	2.23
Experiment 2: rating							
Nongamble vs. gambled loss	.061	.006	.115	2.265	.029	.275	1.66
Nongamble vs. gambled win	.062	−.010	.133	1.746	.089	.267	.68
Gambled loss vs. gambled win	.001	−.055	.057	.032	.975	.004	.17
Experiment 3							
Nongamble vs. gambled loss	−.064	−.115	−.013	−2.537	.015	.314	2.84
Nongamble vs. gambled win	.023	−.032	.079	.842	.405	.109	.24
Gambled loss vs. gambled win	.087	.033	.141	3.244	.002	.448	13.96
Experiment 4							
Nongamble vs. gambled loss	−.026	−.072	.021	−1.113	.272	.131	.30
Nongamble vs. gambled win	.045	.002	.089	2.136	.039	.228	1.31
Gambled loss vs. gambled win	.071	.022	.121	2.913	.006	.374	6.42
Experiment 5							
Nongamble vs. gambled loss	−.029	−.075	.017	−1.257	.216	.156	.35
Nongamble vs. gambled win	.030	−.012	.073	1.444	.157	.162	.44
Gambled loss vs. gambled win	.059	.015	.103	2.703	.010	.310	4.04
*Note.* diff = difference; CI = confidence interval; *g*_av_ = Hedge’s average *g*; BF = Bayes factor. Experiment 1, *df* = 19; Experiments 2–5, *df* = 39.							

**Table C3 tbl10:** Overview of Univariate Analyses to Explore the Effect of the Previous Gamble on p_gamble_ on the Current Gambling Trial in Experiments 1–5

Experiment	*df*1	*df*2	Sum of squares effect	Sum of squares error	*F*	*p*	η_gen_^2^
Experiment 1	2	38	.068	.382	3.396	.044	.039
Experiment 2							
Outcome Trial *n* – 1	2	78	.166	2.205	2.943	.059	.014
Rating	1	39	.011	.238	1.768	.191	.001
Outcome:rating	2	78	.065	.641	3.945	.023	.006
Experiment 3							
Outcome Trial *n* – 1	2	78	.324	2.186	5.775	.005	.030
Casino alternation (CA)	1	39	.001	.525	.094	.761	.000
Outcome by CA	2	78	.006	.589	.370	.692	.001
Experiment 4	2	78	.104	.823	4.925	.010	.023
Experiment 5	2	78	.069	.745	3.630	.031	.017
*Note.* gen = generalized.							

**Table C4 tbl11:** Overview of the Probability of Gambling as a Function of the Median Split (Rating ≤ Median Rating or > Median Rating) and the Preceding Gambling Trial in Experiment 2

		Nongamble		Loss		Gambled win
Statement	≤Median	>Median	≤Median	>Median	≤Median	>Median
“I was pleased with the outcome of the previous trial.”	.59 (.21)	.55 (.21)	.51 (.22)	.49 (.20)	.54 (.22)	.49 (.21)
“I think my chances of winning on the next trial have increased.”	.56 (.22)	.58 (.20)	.47 (.21)	.54 (.20)	.53 (.21)	.50 (.22)
*Note.* *SD* in parentheses.						

**Table C5 tbl12:** Overview of Univariate Analyses to Explore the Effect of the Median Split (Rating: rating ≤ median vs. rating > median) and the Preceding Gambling Trial (Outcome: nongamble, gambled loss, gambled win) on the Probability of Gambling of the Current Gambling Trial in Experiment 2

Statement	*df*1	*df*2	Sum of squares effect	Sum of squares error	*F*	*p*	η_gen_^2^
“Pleased with outcome”							
Outcome Trial *n* – 1	2	66	.175	2.674	2.164	.123	.019
Rating split	1	33	.061	.482	4.164	.049	.007
Outcome:rating split	2	66	.005	.964	.160	.853	.001
“Increased chances of winning”							
Outcome Trial *n* – 1	2	66	.167	2.640	2.093	.131	.019
Rating split	1	33	.030	.561	1.740	.196	.003
Outcome:rating split	2	66	.087	.806	3.548	.034	.010
*Note.* gen = generalized.							

**Table D1 tbl13:** Overview of Choice Latencies for Experiments 1–5 as a Function of the Outcome of the Last Gambling Trial

	Nongamble		Loss		Gambled win	
Experiment	*M*	*SD*	*M*	*SD*	*M*	*SD*
Experiment 1	687	135	672	134	706	150
Experiment 2						
No rating	974	259	939	229	996	273
Rating	980	258	913	228	993	285
Experiment 3						
Casino repetition	662	137	664	168	670	147
Casino alternation	653	123	654	148	678	160
Experiment 4	621	137	605	130	636	139
Experiment 5	612	185	603	178	626	198
*Note.* In Experiments 4 and 5, subjects performed a nongambling task between two successive gambling trials.						

**Table D2 tbl14:** Overview of Planned Comparisons to Explore the Effect of the Previous Gamble on Choice Latencies in Experiments 1–5

Experiment	diff	Lower CI	Upper CI	*t*	*p*	*g*_av_	*BF*
Experiment 1							
Nongamble vs. gambled loss	15	−7	37	1.434	.168	.108	.56
Nongamble vs. gambled win	−19	−53	14	−1.209	.242	.132	.44
Gambled loss vs. gambled win	−34	−64	−4	−2.411	.026	.235	2.32
Experiment 2							
Nongamble vs. gambled loss	50	18	83	3.115	.003	.209	10.26
Nongamble vs. gambled win	−17	−56	21	−.922	.362	.067	.25
Gambled loss vs. gambled win	−68	−102	−34	−4.005	<.001	.276	98.86
Experiment 3							
Nongamble vs. gambled loss	−1	−28	25	−.105	.917	.010	.17
Nongamble vs. gambled win	−17	−45	12	−1.201	.237	.123	.33
Gambled loss vs. gambled win	−16	−36	5	−1.498	.142	.104	.48
Experiment 4							
Nongamble vs. gambled loss	16	0	33	2.031	.049	.121	1.08
Nongamble vs. gambled win	−15	−33	4	−1.620	.113	.106	.56
Gambled loss vs. gambled win	−31	−54	−8	−2.775	.008	.230	4.72
Experiment 5							
Nongamble vs. gambled loss	8	−8	24	1.029	.310	.045	.28
Nongamble vs. gambled win	−14	−33	5	−1.501	.141	.072	.48
Gambled loss vs. gambled win	−22	−43	−1	−2.148	.038	.117	1.34
*Note*. diff = difference; CI = confidence interval; *g*_av_ = Hedge’s average *g*; BF = Bayes factor. Experiment 1, *df* = 19; Experiments 2–5, *df* = 39.							

**Table D3 tbl15:** Overview of Univariate Analyses to Explore the Effect of the Previous Gamble on Choice Latencies on the Current Gambling Trial in Experiments 1–5

Experiment	*df*1	*df*2	Sum of squares effect	Sum of squares error	*F*	*p*	η_gen_^2^
Experiment 1	2	38	11,686	71,002	3.127	.055	.010
Experiment 2							
Outcome Trial *n* – 1	2	78	199,349	947,225	8.208	.001	.013
Rating	1	39	3,474	351,239	.386	.538	.000
Outcome:rating	2	78	10,838	604,614	.699	.500	.001
Experiment 3							
Outcome Trial *n* – 1	2	78	14,065	494,790	1.109	.335	.003
Casino alternation (CA)	1	39	920	110,577	.324	.572	.000
Outcome by CA	2	78	4,329	372,294	.454	.637	.001
Experiment 4	2	78	19,523	143,366	5.311	.007	.009
Experiment 5	2	78	6,740	161,370	1.629	.203	.002
*Note.* gen = generalized.							

## Footnotes

1 In future experiments, we plan to measure EEG when subjects are performing the task. To avoid overlap between visual components associated with the different perceptual events, the minimum delay between the start message and the presentation of the first option was 500 ms.

2 For casino repetition trials, start RT was 596 ms (*SD* = 181) after a gambled loss, 707 ms (*SD* = 250) after a gambled win, and 755 ms (*SD* = 180) after a nongamble. For casino-alternation trials, start RT was 628 ms (*SD* = 225) after a gambled loss, 739 ms (*SD* = 288) after a gambled win, and 789 ms (*SD* = 213) after a nongamble.

3 This would require hundreds of signal trials as the outcome of the previous trial cannot be predicted and some subjects do not gamble very often.

4 Extra analyses (also presented in Supplementary Materials) revealed that gambling per se is not an impulsive action: For many subjects, the latency of the choice response (to gamble or not) was longer for gambles than for nongambles for many subjects.

5 Some have argued that regret is a special (cognitive) form of frustration (Reid, 1986).

## A Combinations of Nongambling and Gambling Options

Overview of the different combinations of options in the experiments (16 in total). Each combination occurred with equal probability.

Expected value = 20:

• 100% certain to win 20 versus 66% certain to win 30
• 100% certain to win 20 versus 50% certain to win 40
• 100% certain to win 20 versus 33% certain to win 60
• 100% certain to win 20 versus 25% certain to win 80

Expected value = 30:

• 100% certain to win 30 versus 66% certain to win 45
• 100% certain to win 30 versus 50% certain to win 60
• 100% certain to win 30 versus 33% certain to win 90
• 100% certain to win 30 versus 25% certain to win 120

Expected value = 40:

• 100% certain to win 40 versus 66% certain to win 60
• 100% certain to win 40 versus 50% certain to win 80
• 100% certain to win 40 versus 33% certain to win 80
• 100% certain to win 40 versus 25% certain to win 160

Expected value = 50:

• 100% certain to win 50 versus 66% certain to win 75
• 100% certain to win 50 versus 50% certain to win 100
• 100% certain to win 50 versus 33% certain to win 150
• 100% certain to win 50 versus 25% certain to win 200

## B Overview of Univariate Analyses

[Table-anchor tbl5][Table-anchor tbl6][Table-anchor tbl7]

## C Probability of Gambling in Experiments 1–5

The combined analysis reported in the main manuscript indicates that subjects gambled less after a gambled win than after a nongamble, and that the probability of gambling was highest after a gambled loss. The *p*_gamble_ analyses for each individual experiment revealed similar numerical differences (Table C1), but the paired *t* tests were not always significant (Table C2). The univariate analyses are presented in Table C3.

For Experiment 2, we also performed a median-split analysis (see the main manuscript for further details). The results are presented in Tables C4–C5. *p*_gamble_ was generally lower for trials with a higher “pleased with outcome” rating than for trials with a lower rating, suggesting that the decision to gamble is influenced by hedonic state. The “increased chances of winning” analysis revealed a significant interaction between the outcome of the previous trial and the “increased chance of winning” rating. After a loss, probability of gambling was higher for high-rating trials than for low rating trials; this difference was statistically significant, *p* = .008, and suggests a cognitive influence on the probability of gambling. For trials following a nongamble or a gambled win, the numerical *p*_gamble_ differences between low- and high-rating trials were not significant (both *p*s > .47).[Table-anchor tbl8][Table-anchor tbl9][Table-anchor tbl10][Table-anchor tbl11][Table-anchor tbl12]

## D Choice Latencies in Experiments 1–5

[Table-anchor tbl13][Table-anchor tbl14][Table-anchor tbl15]
