# Supplementary material for: Winning and Losing: Effects on Impulsive Action
Source: J Exp Psychol Hum Percept Perform. 2016 Nov 3;43(1):147–68. doi: 10.1037/xhp0000284 (PMC5178881; doi:10.1037/xhp0000284)
Supplement: Supplementary file 1 [file GambleLoss_si_160602.pdf]

SUPPLEMENTARY INFORMATION

Winning and losing: Effects on impulsive action

Individual differences in choice RT.

Choice RT was shorter for trials on which subjects selected non-gamble (*M* = 706 ms, *SD* = 223) than for trials on which subjects selected the gamble option (*M* = 738 ms, *SD* = 248), $t(179) = 3.650$, $p < .001$, $g_{av} = 0.132$. However, we observed large individual differences.

For each subject, we calculated a normalized RT difference score: (choice RT gamble minus choice RT non-gamble)/(choice RT non-gamble). Thus, a negative score indicates that the subject selects the gamble proportionally faster than the non-gamble. As can be seen in Figure S1, selecting the gamble took *longer* than selecting the non-gamble for many subjects (i.e. many data points are above the horizontal dashed line). Interestingly, this latency difference correlated strongly with overall p(gamble): the less subjects gambled, the longer it took them to select the gamble compared with selecting the non-gamble, $r(179) = -.62$, $p < .001$ (coefficient robust regression: value = -0.4202, standard error = 0.0391, $t(178) = 10.742$).

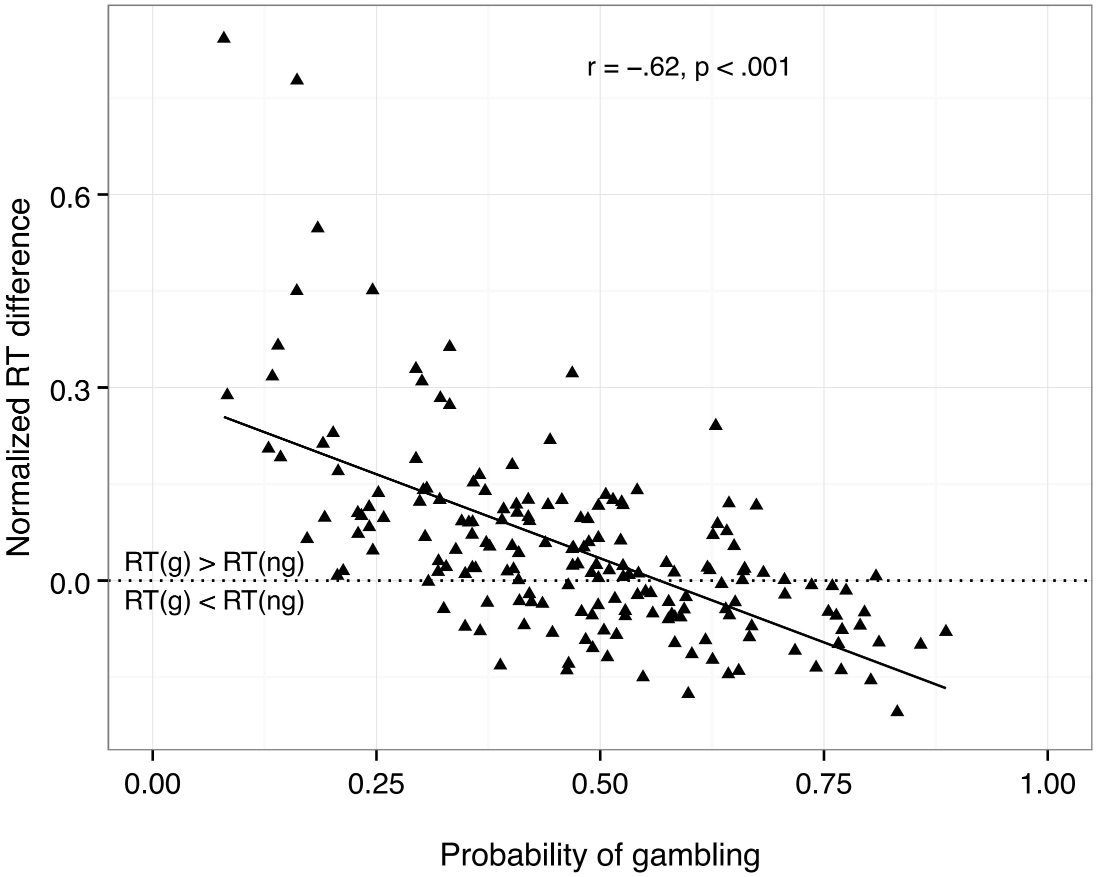

Figure S1: Correlation between overall probability of gambling and the normalized choice RT difference. RT difference = (choice RT gamble minus choice RT non-gamble)/(choice RT non-gamble).

The pattern in Figure S1 is inconsistent with the idea that the decision to gamble is *always* a rash decision or an impulsive act (see also e.g. Losecaat Vermeer, Boksem, & Sanfey, 2014). Stochastic accumulator models of decision making (Smith & Ratcliff, 2004) can offer a parsimonious explanation for the correlation between the choice RT and probability of gambling. Such models assume that decision making involves the accumulation of noisy information until there is enough support for a specific option. The main parameters of the selection process are the response criteria (i.e., how much information is required for an option to be selected), accumulation rate (i.e., how quickly does evidence accumulate), and the starting point (i.e. a priori bias against one or the other choice alternatives; Figure S2). The correlation between p(gamble) and choice RT can be

explained by individual differences in the starting point: when subjects have a bias against gambling (i.e. they are ‘gambling-averse’), the distance between the starting point and the ‘gambling’ boundary will be larger than the distance between the starting point and the non-gambling boundary (Figure S2, right panel). Consequently, the gambling option will be selected less frequently because the accumulated evidence in favor of it is less likely to reach the gambling boundary first. Furthermore, if the gambling boundary is reached after all, the latency of the gambling response will be (on average) longer than the latency of non-gambling responses (Figure S2, right panel). Thus, risk preference can be captured by individual differences in the starting point.

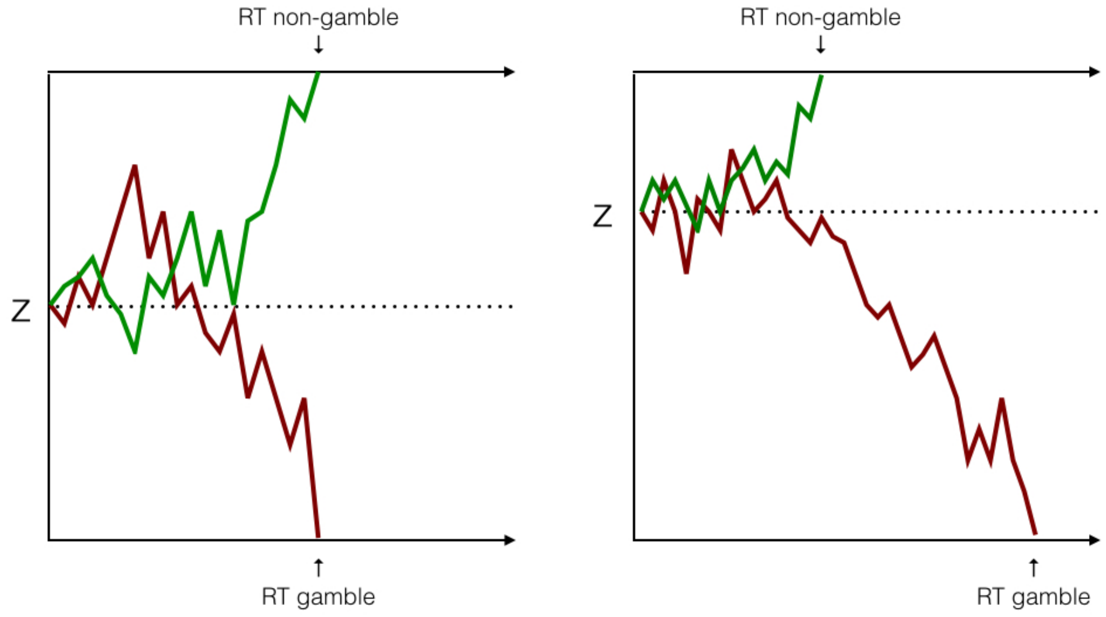

Figure S2: A bias in the starting point (Z) of a sequential decision-making process can explain both the overall probability of gambling and latency differences. The left panel reflects a hypothetical ‘gambling-neutral’ subject; the right panel reflects a hypothetical ‘gambling-averse’ subject.

Does the rating influence start RT of the next trial in Experiment 2?

The p(gamble) analysis for Experiment 2 suggests that the ratings induced a reflective mode: compared with the non-gambling baseline, p(gamble) after a loss decreased on rating trials, but increased (slightly) on no-rating trials.

Here we explored if the ratings also influenced start RT on the next trial (note that the statements were presented after subjects had pressed the start key, so ratings could not influence start RT of the current trial). The results are presented in Figure S3. There was a significant main effect of trial outcome, $F(2,78) = 11.532$, $p < .001$. The main effect of rating on the previous trial, $F(1,39) = 1.223$, $p = .276$, and the interaction, $F(2,78) = 2.234$, $p = .114$, were not significant. Thus, ratings did not influence start RT much.

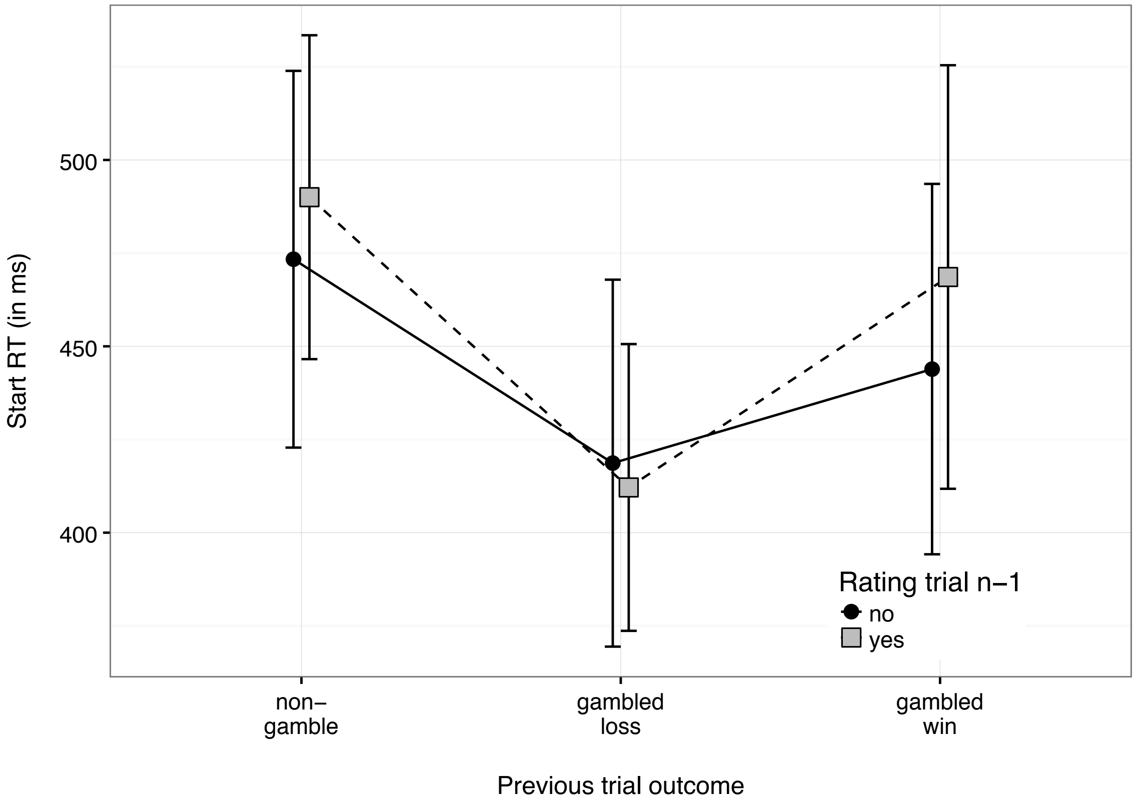

Figure S3: Start RT as a function of the outcome of the previous trial and rating properties of the previous trial (no-rating trial vs. rating trial). Error bars are 95% confidence intervals.

Does the outcome of the non-gambling task influence start RT and p(gamble) on the next trial in Experiments 4 and 5?

In Experiment 4, the outcome of the immediately preceding perceptual decision-making trial (trial *n-1*) influenced performance in the gambling task: subjects started the next gambling trial sooner after an incorrect perceptual decision-making trial (*M* = 541 ms; *SD* = 149) than after a correct trial (*M* = 576; *SD* = 165), *t*(39) = 2.326, *p* = 0.025, g_{av} = .223. This suggests that a negative outcome in difficult perceptual decision-making tasks can also have an 'energizing' effect on behavior (see also e.g. Mikulincer 1988). Choice latencies in the gambling task were also numerically shorter after an incorrect trial (*M* = 613 ms; *SD* = 131 ms) than after an correct trial (*M*= 621 ms; *SD*= 133), but this difference was not statistically significant; *t*(39) = 1.402, *p* = .169, g_{av} = .059. These findings seem inconsistent with previous studies that found post-error slowing. It is possible that the switch design discouraged subjects from making post-error adjustments. However, subjects gambled less after an incorrect perceptual decision-making trial (p(gamble)= .43; *SD* = .18) than after a correct trial (p(gamble)= .46; *SD* = .18); *t*(39) = 2.357, *p* = .024, g_{av} = .153. Thus, error processing in the perceptual decision-making task may have had some influence on choice in the gambling task.

In Experiment 5, subjects alternated between the gambling task and a stop-signal task. The outcome of the immediately preceding stop-signal trial (trial n-1: correct go response, successful stop, unsuccessful stop) did not influence performance in the gambling task much. Subjects started the next gambling trial later after a failed stop (*M =* 779 ms; *SD =* 347) than after a successful stop (*M =* 741 ms; *SD =* 209) or a correct go (*M* = 725 ms; *SD* = 242), but these differences were not statistically significant (Table S1). Nevertheless, the numerical trends are consistent with the idea that subjects increased the priority of the stop

goal after a signal trial (Bissett & Logan, 2011), and this could have counteracted the affective consequences of a negative outcome.

Table S1 shows that p(gamble) were similar for trials that followed a correct go (*M* = .49; *SD* = .17), a successful stop (*M* = .51; *SD* = .18), or an unsuccessful stop (*M* = .48; *SD* = .19), replicating our previous findings (Stevens et al., 2015). Choice latencies were also similar for trials that followed a correct go (*M* = 606 ms; *SD* = 185), a successful stop (*M*= 624 ms; *SD* = 199), or an unsuccessful stop (*M* = 616 ms; *SD* = 190).

Table S1: Overview of planned comparisons to explore the effect of the previous stop-signal trial on performance in the gambling task in Experiment 5. Uncorrected p-values are shown. For all comparisons, *df* = 39.

	diff	***lower CI***	***upper CI***	***t***	***p***	***gav***
Start RT						
Correct vs SR	-55	-122	13	-1.639	0.109	0.184
Correct vs SI	-17	-67	34	-0.668	0.508	0.073
SI vs SR	38	-37	112	1.030	0.309	0.135
P(gamble)						
Correct vs SR	0.014	-0.018	0.047	0.908	0.37	0.079
Correct vs SI	-0.02	-0.054	0.013	-1.232	0.225	0.116
SI vs SR	-0.035	-0.075	0.006	-1.735	0.091	0.188
Choice RT						
Correct vs SR	-10	-30	9	-1.090	0.283	0.055
Correct vs SI	-18	-37	1	-1.965	0.057	0.094
SI vs SR	-8	-31	15	-0.683	0.499	0.040

Note: correct = trials preceded by a correct go (no-signal) trial, SR = trials preceded by a failed stop trial (signal-respond), SI = trials preceded by a successful stop trial (signal-inhibit).

First vs. second half of the experiment

In a final set of analyses, we explored whether the sequential effect of gambling changed throughout the experiment. More specifically, we contrasted performance in the first and second half of the experiment. To increase power, we combined the data of all five experiments again. The relevant descriptive and inferential statistics appear in Tables S2 and S3, respectively. We will focus on the interaction between Trial Outcome and Part only.

Trial outcome had a similar effect on start RT and p(gamble) in the first and second half of the experiment (*p*s > .18; Table S3). We observed a marginally significant interaction between Trial Outcome and Part in the choice RT analysis (p = .051; Table S3). Table S2 shows that choice RTs were longer after a gambled win than after a non-gamble only in the first half of the experiment. Note that choice RTs were shorter after a gambled loss than after a non-gamble in both parts.

Table S2: Overview of the mean start RT, probability of gambling, and choice RT in the gambling task as a function of preceding gambling trial for the first and second part of the experiment (Part 1 vs. Part 2). The data of Experiments 1-5 are combined.

	**Non-gamble**		**Gambled loss**		**Gambled win**	
	M	***SD***	***M***	***SD***	***M***	***SD***
Start RT						
Part 1	718	272	625	251	698	279
Part 2	582	202	486	202	544	228
P(gamble)						
Part 1	0.508	0.202	0.515	0.204	0.432	0.212
Part 2	0.470	0.223	0.480	0.231	0.421	0.247
Choice RT						
Part 1	773	273	743	238	791	290
Part 2	662	209	645	213	663	220

Table S3: Overview of univariate analyses to explore the effect of the previous gamble on performance in the first and second half (Part) of the experiment.

	Df1	***Df2***	***SS1***	***SS2***	***F***	***p***	η^2_{gen}
Start RT							
Outcome	2	358	1685875	5470311	55.165	0.000	0.026
Part	1	179	5517873	7565329	130.556	0.000	0.081
Outcome by Part	2	358	17357	3584990	0.867	0.421	0.000
P(gamble)							
Outcome	2	358	1.102	8.688	22.700	0.000	0.021
Part	1	179	0.211	5.322	7.093	0.008	0.004
Outcome by Part	2	358	0.041	4.340	1.687	0.186	0.001
Choice RT							
Outcome	2	358	207625	2713423	13.697	0.000	0.003
Part	1	179	3387065	4263627	142.199	0.000	0.051
Outcome by Part	2	358	38729	2305363	3.007	0.051	0.001

References

Bissett, P. G., & Logan, G. D. (2011). Balancing cognitive demands: Control adjustments in the stop-signal paradigm. *Journal of Experimental Psychology: Learning, Memory, and Cognition, 37*, 392-404. doi:10.1037/a0021800

Losecaat Vermeer, A. B., Boksem, M. A., & Sanfey, A. G. (2014). Neural mechanisms underlying context-dependent shifts in risk preferences. *NeuroImage, 103*, 355-63. doi: 10.1016/j.neuroimage.2014.09.054

Mikulincer, M. (1988). Reactance and helplessness following exposure to unsolvable problems: The effects of attributional style. *Journal of Personality and Social Psychology, 54*, 679-86.

Stevens, T., Brevers, D., Chambers, C. D., Lavric, A., McLaren, I. P., Mertens, M., . . . Verbruggen, F. (2015). How does response inhibition influence decision making when gambling? *Journal of Experimental Psychology. Applied, 21*, 15-36. doi:10.1037/xap0000039

Smith, P. L., & Ratcliff, R. (2004). Psychology and neurobiology of simple decisions. Trends in Neuroscience, 27, 161-8. doi:10.1016/j.tins.2004.01.006

Verbruggen, F., & Logan, G. D. (2008). Response inhibition in the stop-signal paradigm. *Trends in Cognitive Sciences, 12*, 418-24. doi:10.1016/j.tics.2008.07.005
